# Oxytocin receptor gene methylation as a molecular marker for severity of depressive symptoms in affective disorder patients

**DOI:** 10.1186/s12888-022-04031-w

**Published:** 2022-06-07

**Authors:** Birgit Ludwig, Laura Carlberg, Klemens Kienesberger, Patrick Swoboda, Marleen M. M. Swoboda, Alexandra Bernegger, Romina Koller, Michelle Inaner, Monika Fuxjäger, Melanie Zotter, Nicolas Schmelzle, Birgit Senft, Lisa Meisner, Daniela Fischer-Hansal, Jasmin Huber, Silvia Schoenthaler, Nestor D. Kapusta, Helmuth Haslacher, Martin Aigner, Andreas Weinhaeusel, Siegfried Kasper, Alexandra Schosser

**Affiliations:** 1 Department of Psychiatry and Psychotherapy, Medical University of Vienna, Vienna, Austria; 2 Department of Neurology, Medical University of Vienna, Vienna, Austria; 3 Department of Laboratory Medicine, Medical University of Vienna, Vienna, Austria; 4 Department of Psychiatry and Psychotherapy, Karl Landsteiner University for Health and Science, Tulln, Austria; 5 St. John of God Hospital, Vienna, Austria; 6 Zentren Für Seelische Gesundheit, BBRZ-Med, Vienna, Austria; 7 Health & Environment Department, Molecular Diagnostics Unit, AIT Austrian Institute of Technology, Vienna, Austria; 8 Department of Psychoanalysis and Psychotherapy, Medical University of Vienna, Vienna, Austria; 9 Center for Brain Research, Medical University of Vienna, Vienna, Austria; 10 Faculty of Medicine, Sigmund Freud University, Vienna, Austria; 11 Arbeitsgemeinschaft Für Verhaltensmodifikation, Salzburg, Austria

**Keywords:** Oxytocin Receptor Gene, Methylation, Major depression, Bipolar disorder, Depressive episode, Childhood trauma questionnaire

## Abstract

**Background:**

Oxytocin (OXT) is a neuropeptide and hormone involved in emotional functioning and also seems to play a role in moderating the stress response. Both preclinical and clinical studies point to an increased methylation status of the Oxytocin receptor (OXTR) promoter region with concomitant deficits in social, cognitive and emotional functioning. We hypothesize that methylation levels (%) of the oxytocin receptor promoter region correlate with the severity of depression symptoms and/or with the severity of childhood trauma within this present sample of affective disorder patients.

**Methodology:**

Eight hundred forty six (846) affective disorder patients of Central European origin were recruited at the Department of Psychiatry and Psychotherapy of the Medical University Vienna, the Karl Landsteiner University for Health and Science and Zentren für seelische Gesundheit, BBRZ-Med Leopoldau. Psychiatric assessment included a semi-structured diagnostic interview (Schedules for Clinical Assessment in Neuropsychiatry), the Hamilton Depression Scale and the Childhood Trauma Questionnaire. Concomitantly DNA samples of peripheral blood cells were collected for Multiplexed and Sensitive DNA Methylation Testing.

**Results:**

Our data suggests a positive but not significant association between OXTR promoter Exons 1–3 methylation levels and severity of depression symptoms as well as severity of emotional neglect in affective disorder patients and no association with childhood trauma.

**Conclusions:**

Our findings contribute to elucidate the role of OXTR in affective disorders, but further longitudinal studies in particular are necessary to broaden the current state of knowledge.

**Supplementary Information:**

The online version contains supplementary material available at 10.1186/s12888-022-04031-w.

## Background

Oxytocin (OXT) is a neuropeptide and hormone involved in emotional functioning and also seems to play a role in moderating the stress response. Oxytocin receptors are synthesized by the Oxytocin Receptor (OXTR) gene and expressed both in the brain and peripheral organs. The OXTR gene spans 17 kilobytes (kb) and contains 4 exons and 3 introns. While exons 1 and 2 correspond to non-coding regions, exons 3 and 4 encode the amino acids of the OXTR gene. Molecular-wise it is a class I G-protein coupled transmembrane receptor, facilitating the oxytocin pathway [1].

One of the most established epigenetic mechanisms is DNA methylation. High levels of DNA methylation are commonly known to decrease mRNA expression, differential methylation is one of the known mechanisms to regulate gene expression [2]. The functional significance of OXTR DNA methylation has been supported by both rodent and clinical data. Researchers showed that the mouse oxytocin receptor gene was epigenetically regulated by DNA methylation of its promoter and they also found that specific CpG sites were differentially methylated between distinct murine brain regions expressing different levels of OXTR mRNA [3, 4].

Findings in psychiatric disorders in general point to an increased methylation status of the OXTR promoter region with decreased peripheral gene expression in individuals afflicted with depression, anxiety or situations of increased stress [5, 6].

Preclinical findings suggest that long-term isolation down-regulates OXTR mRNA transcription and contributes to the development of depression in isolated mice – which might be attenuated through intracentral amygdala injection of OXT [7]. In a rodent model of postpartal depression, using a social stress paradigma during lactation, a significant decrease in oxytocin mRNA expression in the medial amygdala was found [8]. More recent preclinical findings in female rodents suggest that chronic social defeat stress might increase the levels of anxiety and depression via a reduction in oxytocin projections and the oxytocin receptor level in the nucleus accumbens [9]. Since Oxytocin is involved in social bonding and attachment to others, it has been hypothethized that childhood trauma might lead to dysfunctions in the OXTR system [10]. Clinical data suggests that mothers with early trauma (loss of a parent or sexual abuse in childhood) had lower serum OXTR mRNA than no-trauma mothers [11]. OXTR protein expression levels were significantly decreased in women having suffered from exposure to childhood trauma, and there was a also a significant negative correlation between OXTR protein expression levels and the Childhood Trauma Questionnaire (CTQ) score [12]. A Japanese study focusing on children with and without a history of childhood maltreatment compared salivary OXTR methylation levels showing higher CpG 5,6 methylation of the maltreated children [13]. Smearman et al. [14] attempted to unveil the associations between early life adversities, DNA methylation of OXTR gene and adult psychiatric symptoms. Child abuse was associated with higher methylation of two CpG sites, yet did not survive correction for multiple testing. Childhood abuse interacted with CpG methylation in 3 of 5 tested CpG islands to predict depression in adulthood [14].

In a study including 309 African American men, childhood adversity was not directly associated with elevated OXTR methylation but could be explained by the factor of socioeconomic precarity [15]. Several studies also suggest a link between OXTR and the attachment system, OXTR might not only mediate the parent–child attachment formation and maintenance, oxytocin might also interact with adult attachment style [16]. Since childhood adversity is highly associated with the occurrence of affective disorders in adults, multiple studies hypothethized that adults with affective disorders would also show a dysfunction in the OXTR system.

There is evidence suggesting that both Major Depression Disorder (MDD) and Bipolar Disorder (BPD) patients show increased expression of OXTR mRNA in the dorsolateral prefrontal cortex compared to patients with schizophrenia and controls [17].

### Previous publications report inconclusive results regarding the implication of OXTR

promoter methylation in affective disorders but point in general to an increase of OXTR gene promoter methylation in depressed individuals [5, 6].

Rainer et al. [18] reported significantly decreased methylation of Exon I in the oxytocin receptor promoter region in depressed female vs. healthy female controls [18]. A similar study investigating postmenopausal women with anxiety and depression, found oxytocin receptor methylation to be increased in a subgroup of rs53576 AA carriers when compared to healthy age-matched female controls [19]. Another study focusing on postpartal depressive women found increased DNA methylation of the OXTR only in individuals with a rs53576 GG genotype [20].

Based on the above presented findings in the literature, we hypothethize that methylation of OXTR gene promoter region is positively correlated with severity of history of childhood trauma and current severity of depression in a large cohort of affective disorder patients. As a secondary outcome, we hypothethize that the positive association between a history of childhood trauma (CTQ score) and current depressive symptoms as an adult (HAMD score) is mediated by OXTR promoter region upregulation.

## Methods

### Participants

A total of 846 unrelated in- and outpatients with affective disorders were recruited at three study sites in Vienna and the surrounding area. A total of 382 patients were recruited at the Department of Psychiatry and Psychotherapy of the Medical University Vienna, 67 patients Karl Landsteiner University for Health and Science and 397 patients at the Zentrum für seelische Gesundheit, BBRZ-Med Leopoldau in the context of the Austrian Science Funds (FWF) funded study “VieSAD” (“Vienna Study on Genetics of Suicidal Behavior in Affective Disorders”, KLI°220). The investigation was carried out in accordance with the latest version of the Declaration of Helsinki and approval for the study was obtained from the Ethical Committee of the Medical University of Vienna (EK 2013/2013) and the federal state of Lower Austria (GS4- EK-4/181/2012).

Patients of Central European origin aged from 18 to 65 years were included if they were diagnosed with either bipolar disorder (BPD) or major depressive disorder (MDD) as defined by ICD-10 and/or DSM-IV criteria. Exclusion criteria were mood incongruent psychotic symptoms or lifetime history of schizophrenia, primary organic disease, primary substance abuse, pregnancy and breastfeeding. Diagnosis was affirmed by performing detailed clinical examination (SCAN– Schedules for Clinical Assessment in Neuropsychiatry[21]). Additionally, a self-report scale was applied to screen for traumatic events in childhood, the CTQ-SF (short form of the Childhood Trauma Questionnaire) [22]. Cut-off scores were used as defined by Bernstein & Fink [23]. In order to screen for acute affective states, the HAMD (Hamilton Depression Scale [24]) was applied when blood for genotyping was drawn. HAMD cut-off scores are referenced in the NICE guidelines (2019). Comorbidities were monitored, as well as weight, height and Body Mass Index. In a face-to-face interview, patients were informed about the study and signed a written consent form. Interrater reliability was guaranteed by extensive interview training, following Good Clinical Practice criteria. Biomaterial was processed and stored at the MedUni Wien Biobank facility in an ISO 9001-certified environment according to standard operating procedures published previously [25].

### Statistical analyses

All statistics were conducted using the statistical software SPSS 27.0 (IBM, Armonk USA) and ‘R 3.4.2’ (cran.r-project.org/). Continuous data were presented as mean and standard deviation, respectively with confidence intervals. Categorical data were given as counts and percentages. Normal distribution of the variables was tested by Shapiro–Wilk test. None of the tested variables were normally distributed (OXTR methylation: W = 0.129, *p* < 0.001, *n* = 748; HAMD score: W = 0.994, *p* = 0.002, *n* = 748; CTQ score: W = 0.921, *p* < 0.001, *n* = 748). Consecutively, non-parametric testing was applied for all variables. Differences between more than two groups were assessed by means of the Kruskall-Wallis H Test. For non-parametric correlation data, Kendall-Tau-b correlation coefficient was calculated. All test results were interpreted two-tailed with a significance level established at *p* ≤ 0.05.

Power analyses were performed using the software R (Version 1.1.456) with the “R” package “pwr”. The appropriate sample size was estimated along the lines of Cohen (1988) (pwr.t.test(*n *= NULL, d = 0.5, sig.level = 0.05, power = 0.80, type = c("two.sample"), alternative = c("two.sided")). Assuming a medium clinical effect size of d = 0.5, one sample, and accepting an α-error of 0.05 and a β-error of 0.2 (power = 0.8), those effects would be detectable at a sample size ≥ 34 per group. For ANOVA comparing 5 samples, assuming a medium effect of f = 0.4, accepting an α-error of 0.05 and a β-error of 0.2 (power = 0.8), those effects would be detectable at a sample size ≥ 16 per group. For the mediation analysis, we used the PROCESS macro (Version 4.0) by SPSS Version 27.0 (IBM, Armonk USA). A bootstrap approach with 5,000 bootstrap samples was used for the test of statistical significance of the indirect effect.

### Methylation analysis

Epigenetic methylation analyses were performed using the MSRE (methylation-sensitive restriction enzyme) – qPCR (quantitative polymerase chain reaction) approach [26]. For methylation analyses, based on previous literature, the CpG covering part of OXTR Exons 1–3 promoter region (chr3:8,769,499–8,769,620,hg 38) with a size of 121 bp (PCR primers—forward: gctggggctgaggctgcactatc and reverse: cccatttgttaaggctctgggaccaa), containing a total of 12 CpG sites; of those 6 CpGs were covered by the methylation sensitive restriction enzymes AciI 32, AciI 42, AciI 56, Hin6i 58, HpaII 70, and AciI 75. An additional figure file provides more details [see Additional file 1].

Digestion of all samples and parallel incubation without digestion were performed the same day. 2 µl digested/undigested DNA was used for the following qPCR protocol: hotstart activation with 95 °C for 5 min, followed by amplification (45x) with 95 °C for 40 s, 65 °C for 40 s, 72 °C for 1 min 20 s, followed by the final extension step with 72 °C for 7 min and cool-down phase with 4 °C. Raw data of methylation analyses (Ct- and Tm-values) were calculated. Methylation status in % was calculated relatively to the reference values of undigested DNA samples applying the following formula:

$$\mathrm{\% }methylation = sample digested [ng] / sample undigested [ng] * 100\mathrm{\%}$$

The according values were reported as percentage of methylated reference (PMR) values.

## Results

In total, 846 patients were recruited. Methylation data was available for a total of 814 patients. Both HAMD data and methylation data were available for a total of 757 patients. Both complete CTQ data and methylation data were available for 751 patients. HAMD, CTQ and methylation data were available for a total of 748 patients.

The mean age of the participants was 44.94 years (SD =  ± 12.9, n = 748), 489 of them were women (65,4%) and 259 of them were men (34.6%). A total of 656 patients (87.7%) were included with a diagnosis of Major Depression Disorder, a total of 92 patients (12.3%) were included with a diagnosis of Bipolar Disorder. Regarding axis II comorbities a total of 110 patients (14.7%) had a personality disorder diagnosis as well. Severity of depression symptoms in our patient population is further described in Table 1, severity of childhood trauma within our patient population is further described in Table 2.

**Table 1:** The mean score of the 17-item Hamilton Rating Scale for Depression questionnaire was 15.9 (SD =  ± 6.7) in our patient population. SD = standard deviation

***HAMD Scores***	**No Symptoms (0–7)**	**Mild Symptoms (8–13)**	**Moderate Symptoms (14–18)**	**Severe Symptoms (19–22)**	**Very severe Symptoms > 23**
*Percentage (%)*	10.7	25.4	32.2	15.8	15.9
*n*	80	190	241	118	119

**Table 2:** The overall mean total Childhood Trauma Questionnaire score of this sample was 50.04 (SD ± 20.6). SD = standard deviation

*CTQ Scores*	None to minimal trauma	Mild to moderate trauma	Moderate to severe trauma	Severe to extreme trauma	Mean Scores (SD)
*Emotional Abuse*	285 (38.1%)	147 (19.7%)	93 (12.4%)	223 (29.8%)	12.02 (± 6.22)
*Physical Abuse*	497 (66.4%)	73 (9.8%)	59 (7.9%)	119 (15.9%)	7.96 (± 4.61)
*Sexual Abuse*	561 (75%)	27 (3.6%)	50 (6.7%)	109 (14.6%)	7.27 (± 4.90)
*Emotional Neglect*	241 (32.2%)	174 (23.3%)	105 (14%)	228 (30.5%)	13.83 (± 6.34)
*Physical Neglect*	348 (46.5%)	138 (18.4%)	118 (15.8%)	144 (19.3%)	8.96 (± 4.14)

### Methylation analysis

To examine the methylation status of OXTR gene promoter region, the MSRE – qPCR approach was performed. Since there was no significant difference between male and female affective disorder patients in the PMR values of the OXTR gene in the present sample (f = 0.29 ± 0.03, m = 0.45 ± 0.16; U = 0.461; *p* = 0.65, *n* = 748, Mann–Whitney-U-test), all further analyses were exercised for the entire sample. Since there was no significant correlation between age and the PMR values of the OXTR gene as inferred from the Kendall-Tau-b correlation coefficient (τ = 0.032, *p* = 0.20, *n* = 748), further analyses were not corrected for age.

**Fig. 1:**
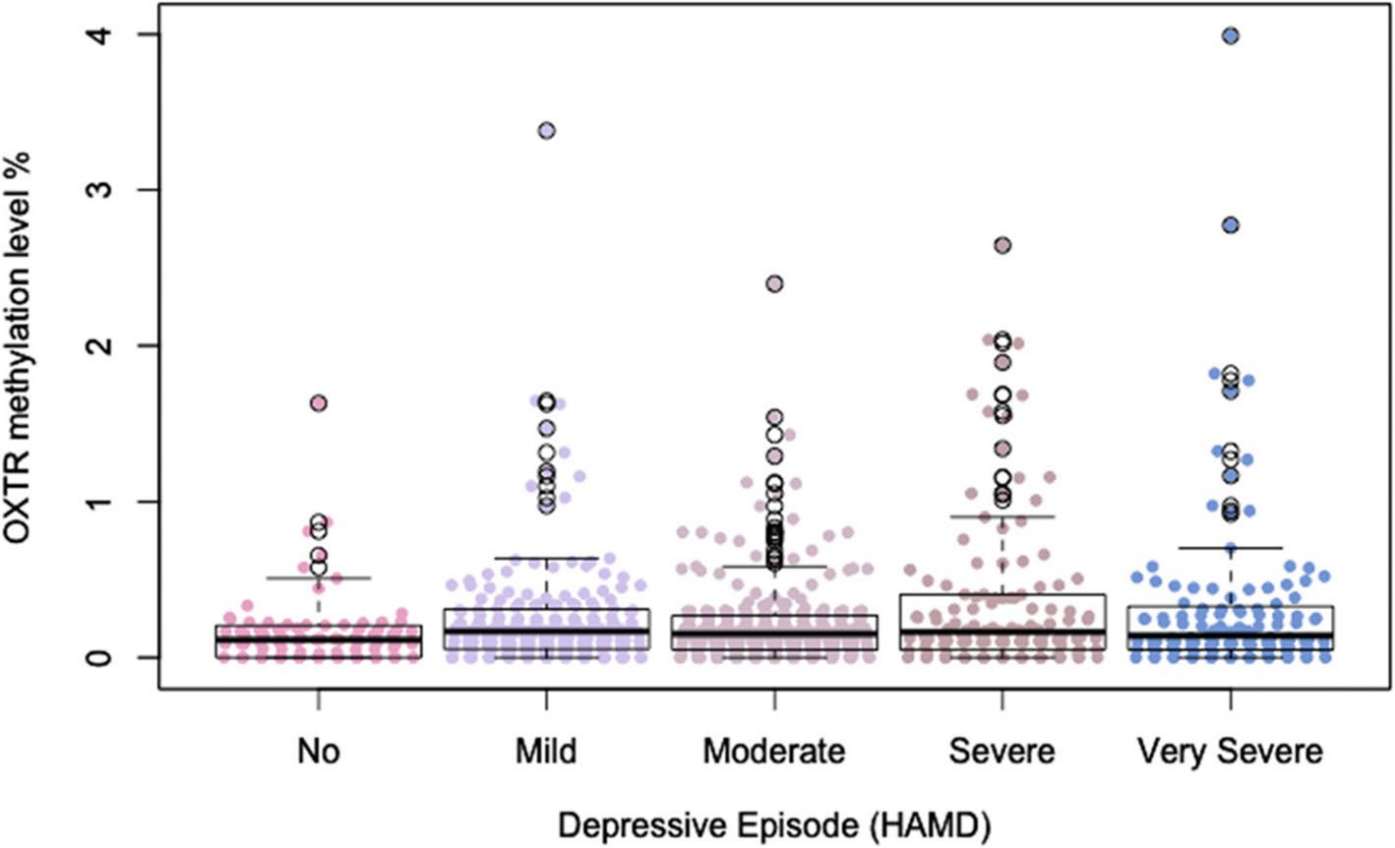
Box plots presenting HAMD score categories, differentiating between no symptoms (*n* = 80), mild symptoms (*n* = 190), moderate symptoms (*n* = 241), severe symptoms (*n* = 118) and very severe depressive symptoms (*n* = 119) based on the percentage of methylation ratio (PMR) values of OXTR gene promotor methylation. The analysis resulted in no significant differences in between categories based on the PMR values of OXTR gene promotor methylation (W = 6.306; *p* = 0.18, *n* = 748). Outliers (detected with Z-transformation) were removed from the graph for a better visualization of the results (whereas within the statistical analyses no outliers were removed)

One-way ANOVA with Kruskall-Wallis was applied on HAMD score categories, differentiating between no symptoms (*n* = 80), mild symptoms (*n* = 190), moderate symptoms (*n* = 241), severe symptoms (*n* = 118) and very severe depressive symptoms (*n* = 119). The analysis resulted in no significant differences in between categories based on the PMR values of OXTR gene promotor methylation (W = 6.306; *p* = 0.18, *n* = 748) (Fig. 1).

A one-way ANOVA with Kruskall-Wallis comparing oxytocin gene promoter methylation (%) between none to low trauma exposure, low to moderate trauma exposure, moderate to severe trauma exposure and severe to extreme trauma exposure within the CTQ subscales showed no significant differences in any of the subscales (Table 3).

**Table 3:** CTQ subscales with classification according to Bernstein comparing mean PMR values of the OXTR promotor region (Oneway-ANOVA Kruskall-Wallis with pair-wise comparison)

**CTQ-Subscale**	*H*	*p*-value	*n*
Emotional Abuse	4.144	0.25	748
Emotional Neglect	4.507	0.21	748
Physical Abuse	5.028	0.17	748
Physical Neglect	0.553	0.91	748
Sexual Abuse	5.324	0.15	748

An additional figure file shows this in more detail [see Additional file 2].

In this study investigating affective disorder patients, there was also a highly significant positive correlation (τ = 0.15, *p* < 0.0001, *n* = 788) between the total Hamilton score and the total CTQ-score as we already reported previously [27]. An additional figure file shows this in more detail [see Additional file 3].

**Fig. 2:**
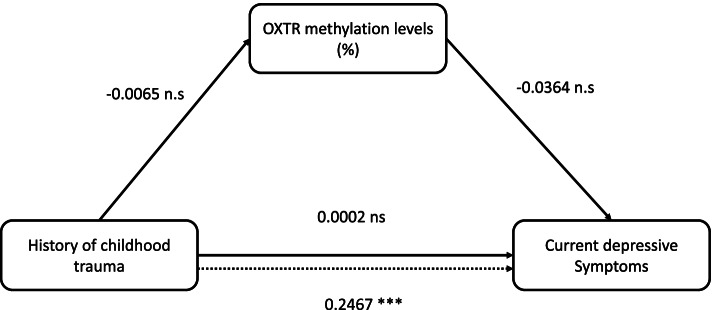
Representation of a mediation model with childhood trauma (total CTQ-score) as dependent variable, depressive symptoms (total HAMD score) as independent variable and OXTR methylation levels (%) als mediator variable. *** *p* < 0.001. ns, not significant

We conducted a mediation analysis to find out whether the PMR values of OXTR gene promotor methylation influence the association of severity of history of childhood trauma and severity of depressive symptoms. Standardized results of the analyses are displayed in Fig. 2. The modell is significant (*R* = 0.2467, F = 48.3363, *p* < 0.001) but only accounts for 6.1% (R^2^ = 0.0609) of the variance of the outcome variable (current depressive symptoms). There is a significant direct effect that predicts current depressive symptoms by means of childhood trauma (B = 0.08, t = 6.945, *p* < 0.001). The indirect effect between childhood trauma and depressive symptoms mediated via CTQ score was not significant; the results were -0.004 for the lower and 0.0037 for the upper confidence interval limit (95%). Therefore, the indirect effect via OXTR methylation levels (%) was not significant and small regarding the effect size (*R* = 0.0001).

## Discussion

The aim of the current study was to investigate a possible association between the methylation status of the OXTR gene promoter region and severity of depression symptoms as well as early life adversities.

Our main interest was to determine if the OXTR promoter region methylation status was associated with the severity of depressive symptoms in affective disorder patients. Using an established depression questionnaire at the time of blood draw, we aimed to correlate biological markers with clinical parameters from standardized questionnaires in a timely manner. The present results suggest that severity of depression in affective disorders is not significantly associated with PMR values of OXTR promoter region. The positive but not significant association between severity of depression and PMR levels is visually recognizable on Fig. 1. To the best of our knowledge, this study is the first one to examine the severity of depressive symptoms in relation to the methylation status of OXTR promoter region. Thus, we followed a transdiagnostic approach, priorizing the affective state versus the diagnosis Further studies testing these hypotheses are duly needed. Another interest of the present study was to determine if the methylation status of OXTR promoter region was associated with early life adversities. Again, a standardized questionnaire (CTQ) was used to establish if and to what extent these affective disorder patients had experienced childhood trauma. Overall, there was no significant association between the CTQ score subscales and PMR values. Interestingly, in the association between OXTR methylation levels and the CTQ scores of the subscale Emotional Neglect visually ressembles the pattern of distribution of the HAMD-OXTR figure (Supplementary Fig. 6). One could hypothethize, that emotional neglect in childhood leads to an increased methylation of the OXTR promoter region with concomitant decrease of peripheral OXTR and the occurrence of depressive episodes later in life. Further studies are needed to test this hypothesis. CpG islands tend to be unmethylated and higher PMR values of methylation in CpG islands within gene promoter regions tend to correlate with a repression of gene (and protein) expression in the corresponding tissue. The already mentioned studies by Krause et al. [12] and Light et al. [11] showed a negative association between OXTR protein/gene expression and CTQ-score, which aligns with the trend in our findings. A secondary outcome variable was the supposed effect of OXTR methylation mediating the association between childhood trauma and depressive symptoms. In a mediation model, this indirect effect could not be confirmed. There might be other epigenetic mechanisms mediating this association as a biological correlate. Another explaination might be the non-specific analysis of the OXTR promoter region and/or the statistical approach used in this study. In Smearman et al.’s [14] study, specific CpG islands were analyzed regarding their moderating and mediating effect of childhood trauma and depression; 3 out of 5 CpG islands might have a moderating effect but none showed a significant mediating effect [14].

Several publications suggest sex differences in the methylation status of OXTR promoter region [28, 29], but in our cohort (748 analyzed samples) these findings could not be replicated. Regarding the comparison with Nawjin et al.’s study it must be noted, that they found sex-specific methylation patterns comparing female and male Posttraumatic Stress Disorder (PTSD) patients (total of 62 samples) in OXTR exon 3. Our study included a total of 808 analyzed samples of the exon 1 OXTR gene promoter region in diagnosed affective disorder patients: no significantly different PMR values were found between affected men and women. The second referenced study also has important differences with our study: their patients were diagnosed with psychotic disorders (total of 242) and again a different region within the OXTR promotor was analyzed [29]. Recent research suggests that females might be more sensitive to the impact of early life adversities on OXTR methylation [30]. In the same study they also found sex differences in a cohort of men and women without any previous diagnosis. Women had significantly lower DNA methylation in the promoter region than males, but higher DNA methylation in two analyzed Intron regions [30]. Although sex differences were not significant in our sample, we also found women to have lower DNA methylation in the promoter region of OXTR gene than men.

Limitations of this study include, that CpG islands were not specified but the methylation of 6 CpGs was analyzed as a whole. Specific CpG island effects and potential associations with depressive symptoms and a history of childhood trauma might have been overlooked, since we only calculated with an average of the above-mentioned 6 CpG islands. More specific analyses as done by Fujisawa et al. [13] or Smearman et al. [14] might have resulted in more precise data. Another limitation is the retrospective analysis of data. We included and interviewed all affective disorder patients meeting the inclusion criteria, not based on their current severity of depression. Consequently, when comparing the groups of different severity of depression, the individual group size differs, but in total the divided group sizes were still bigger than the total of included samples in most of the studies referenced in this manuscript. Another limitation of this study is the self-report aspect and the retrospective aspect of the CTQ-score, patients might have trouble remembering or reporting experiences that happened in their childhood. Most patients included in this study were treated with medication and/or supportive counselling. Leaving patients with current affective episode untreated would have raised severe ethical concerns. The effect of (different) treatment is a confounding factor in this study.

One of the clear strengths of this study is the precise phenotypic definition of our sample, which results in a highly homogeneous sample (namely affective disorder patients only) and allows us to prevent interference of the confounding affective disorders phenotype. Another strength of this study is the large sample size of 748 analyzed samples of affective disorder patients, compared to previous clinical methylation studies.

## Conclusions

In conclusion, the herein presented results could not support the hypothesis that increased methylation of OXTR gene promoter region and supposedly downregulation of OXTR in peripheral tissues is associated with a higher severity of depressive symptoms in affective disorder patients. Neither could we show a significant association between higher methylation levels of OXTR gene promoter region and higher CTQ scores within the subscale. Neither could we prove that OXTR methylation mediates the effect of childhood trauma on depressive symptoms. But as shown before, early life adversities and depressive symptoms significantly correlate within our sample [27].

Our findings contribute to elucidate the role of OXTR in affective states, but further longitudinal and transdiagnostic studies in particular are necessary to broaden the current state of knowledge.

## Supplementary Information

**Additional file 1.** 

**Additional file 2.**

**Additional file 3.**

## Data Availability

The datasets supporting the conclusions of this article are available in the repository [https://mfr.osf.io/render?url=https%3A%2F%2Fosf.io%2Fqwmkj%2Fdownload].

## Abbreviations

BPD: Bipolar Affective Disorder
CTQ: Childhood Trauma Questionnaire
HAMD: 17-Item Hamilton Rating Scale for Depression
MDD: Major Depressive Disorder
MSRE: Methylation-sensitive restriction enzyme
OR: Odds Ratio
OXT: Oxytocin
OXTR: Oxytocin Receptor
PMR: Percentage of Methylation Ratio
PTSD: Posttraumatic Stress Disorder
SCAN: Schedules for Clinical Assessment in Neuropsychiatry
VieSAD: Vienna Study on Genetics of Suicidal Behavior in Affective Disorders
