# Supplementary material for: Oxytocin receptor gene methylation as a molecular marker for severity of depressive symptoms in affective disorder patients
Source: BMC Psychiatry. 2022 Jun 7;22:381. doi: 10.1186/s12888-022-04031-w (PMC9172116; doi:10.1186/s12888-022-04031-w)
Supplement: Supplementary file 1 — Additional file 1. [file 12888_2022_4031_MOESM1_ESM.pdf]

Additional File 1

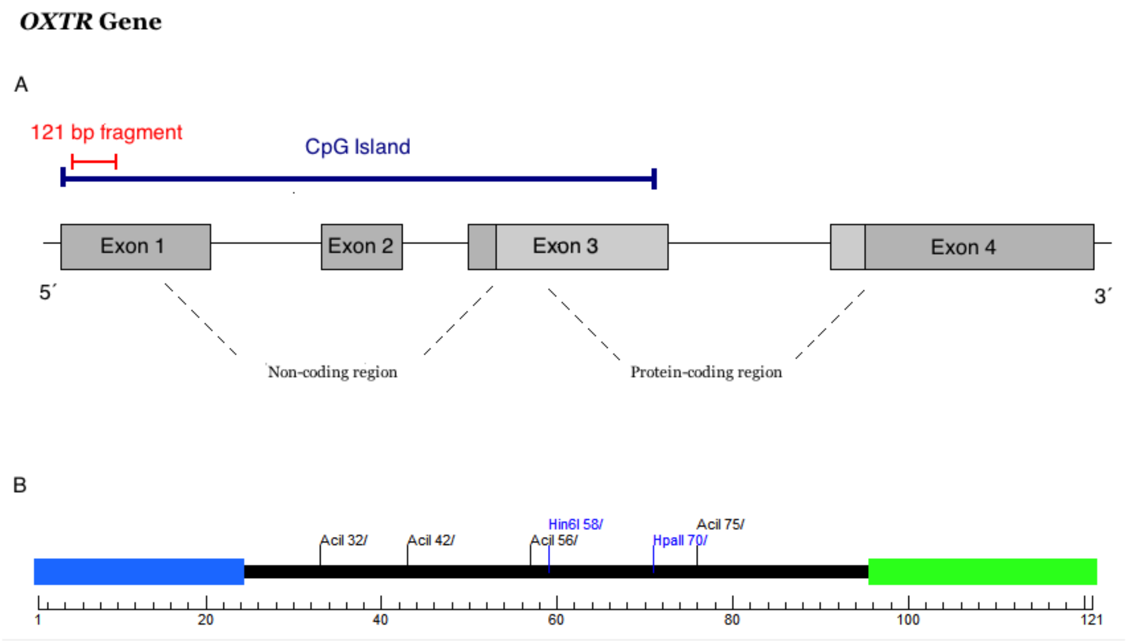

Supplementary Figure 1: Panel A (top) shows the genomic organization of the OXTR gene, which is located on chromosome 3p25-3p26.2 and contains four exons spanning over 17kb. Panel B (bottom) shows the region within the CpG island spanning 121 base pairs that was investigated with regard to differential methylation. The selected regions contains a total of 12 CpG sites; of those 6 CpGs were covered by the methylation sensitive restriction enzymes AciI 32, AciI 42, AciI 56, Hin6i 58, HpaII 70, and AciI 75.
