# Supplementary material for: Oxytocin receptor gene methylation as a molecular marker for severity of depressive symptoms in affective disorder patients
Source: BMC Psychiatry. 2022 Jun 7;22:381. doi: 10.1186/s12888-022-04031-w (PMC9172116; doi:10.1186/s12888-022-04031-w)
Supplement: Supplementary file 2 — Additional file 2. [file 12888_2022_4031_MOESM2_ESM.pdf]

Additional File 2

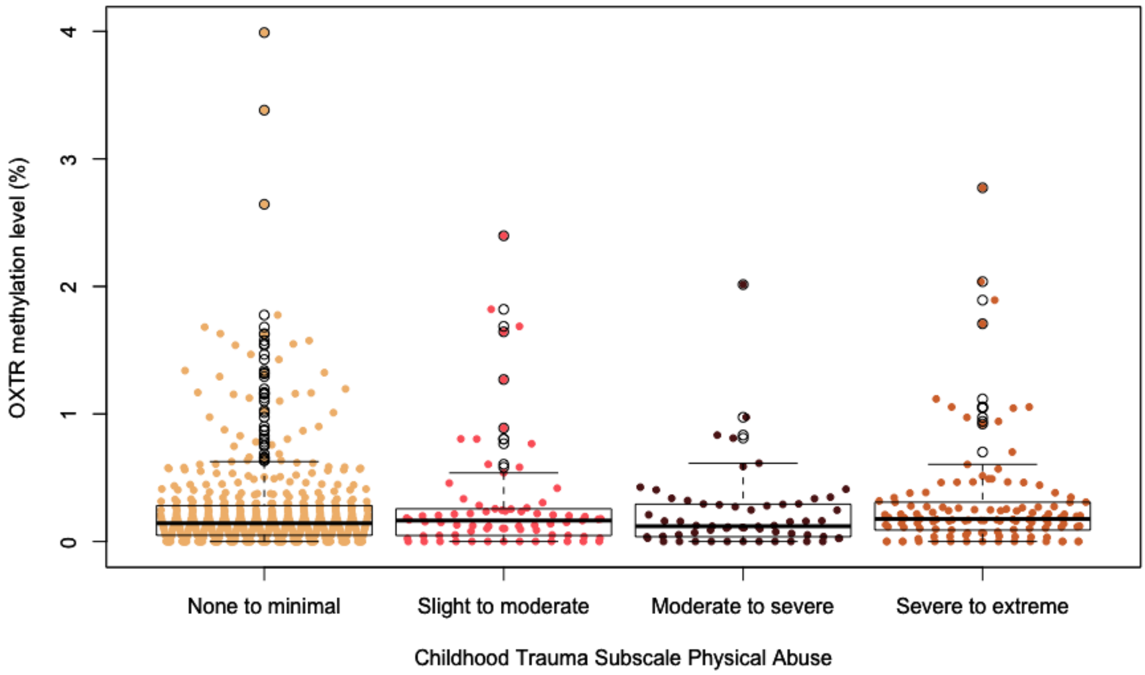

Supplementary Figure 2: Box plots showing oxytocin gene promoter methylation (%) in none to low trauma exposure, low to moderate trauma exposure, moderate to severe trauma exposure and severe to extreme trauma exposure within the CTQ subscale Physical Abuse. A Kruskall-Wallis nonparametric analysis of variance with pair-wise comparison comparing oxytocin gene promoter methylation (%) between none to low trauma exposure, low to moderate trauma exposure, moderate to severe trauma exposure and severe to extreme trauma exposure within the CTQ subscale Physical Abuse, showed no significant difference ($F = 1.92$, $p = 0.13$, $n = 748$) between the categories. Outliers (detected with Z-transformation) were removed from the graph for a better visualization of the results (whereas within the statistical analyses no outliers were removed).

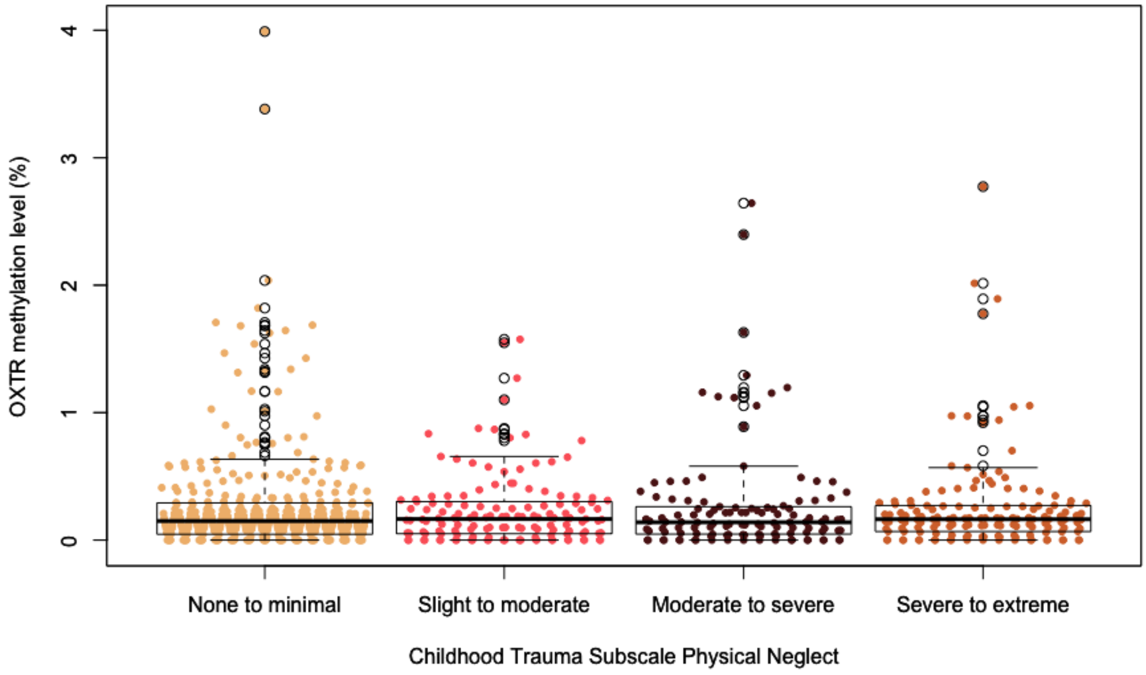

Supplementary Figure 3: Box plots showing oxytocin gene promoter methylation (%) in none to low trauma exposure, low to moderate trauma exposure, moderate to severe trauma exposure and severe to extreme trauma exposure within the CTQ subscale Physical Neglect. A Kruskall-Wallis nonparametric analysis of variance with pair-wise comparison comparing oxytocin gene promoter methylation (%) between none to low trauma exposure, low to moderate trauma exposure, moderate to severe trauma exposure and severe to extreme trauma exposure within the CTQ subscale Physical Neglect, showed no significant difference (H=0.553, p = 0.91, n = 748) between the categories. Outliers (detected with Z-transformation) were removed from the graph for a better visualization of the results (whereas within the statistical analyses no outliers were removed).

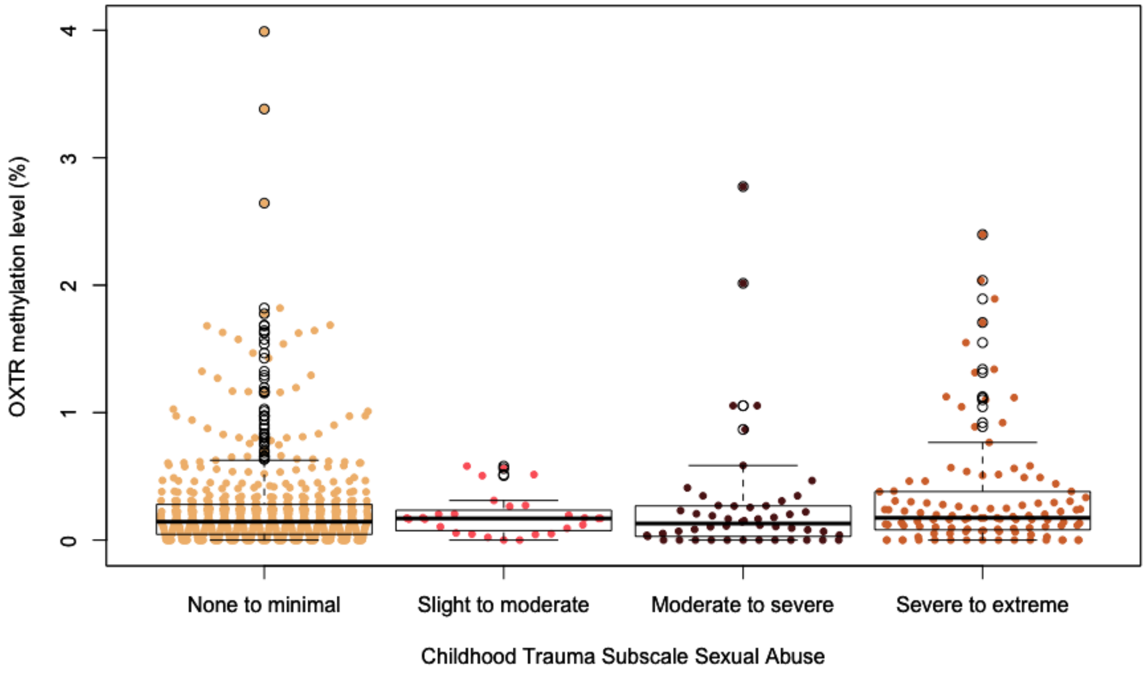

Supplementary Figure 4: Box plots showing oxytocin gene promoter methylation (%) in none to low trauma exposure, low to moderate trauma exposure, moderate to severe trauma exposure and severe to extreme trauma exposure within the CTQ subscale Seuxal Abuse. A Kruskall-Wallis nonparametric analysis of variance with pair-wise comparison comparing oxytocin gene promoter methylation (%) between none to low trauma exposure, low to moderate trauma exposure, moderate to severe trauma exposure and severe to extreme trauma exposure within the CTQ subscale Sexual Abuse, showed no significant difference (H=5.324, p = 0.15, n = 748) between the categories. Outliers (detected with Z-transformation) were removed from the graph for a better visualization of the results (whereas within the statistical analyses no outliers were removed).

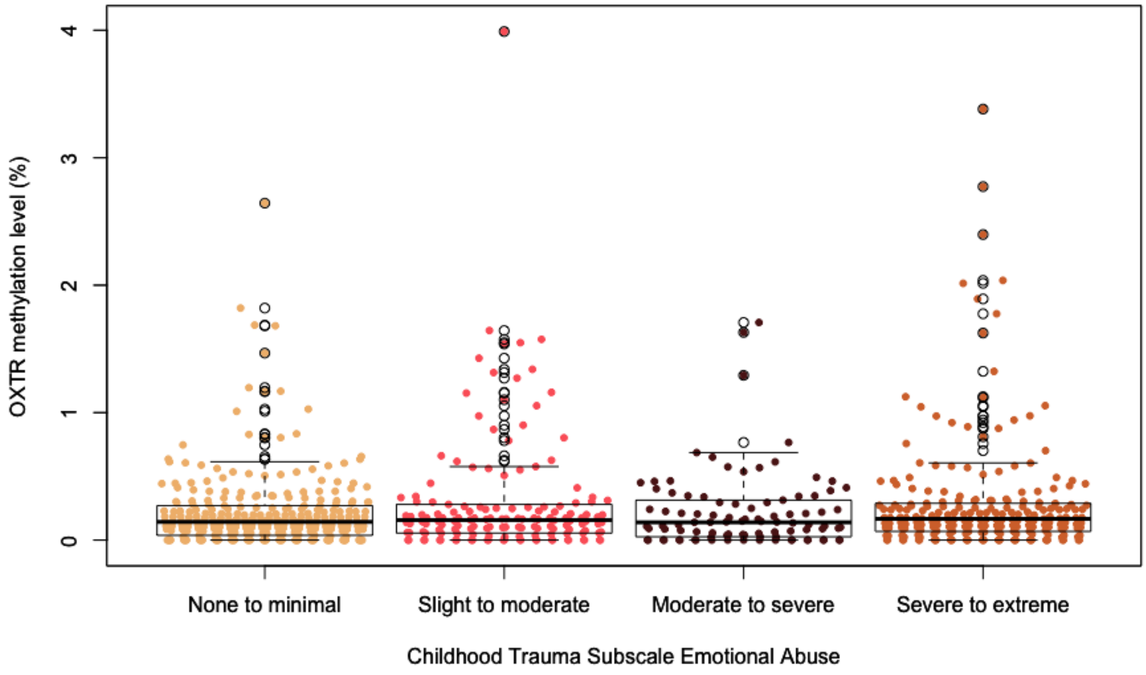

Supplementary Figure 5: Box plots showing oxytocin gene promoter methylation (%) in none to low trauma exposure, low to moderate trauma exposure, moderate to severe trauma exposure and severe to extreme trauma exposure within the CTQ subscale Emotional Abuse. A Kruskall-Wallis nonparametric analysis of variance with pair-wise comparison comparing oxytocin gene promoter methylation (%) between none to low trauma exposure, low to moderate trauma exposure, moderate to severe trauma exposure and severe to extreme trauma exposure within the CTQ subscale Emotional Abuse, showed no significant difference (H=4.144, p = 0.25, n = 748) between the categories. Outliers (detected with Z-transformation) were removed from the graph for a better visualization of the results (whereas within the statistical analyses no outliers were removed).

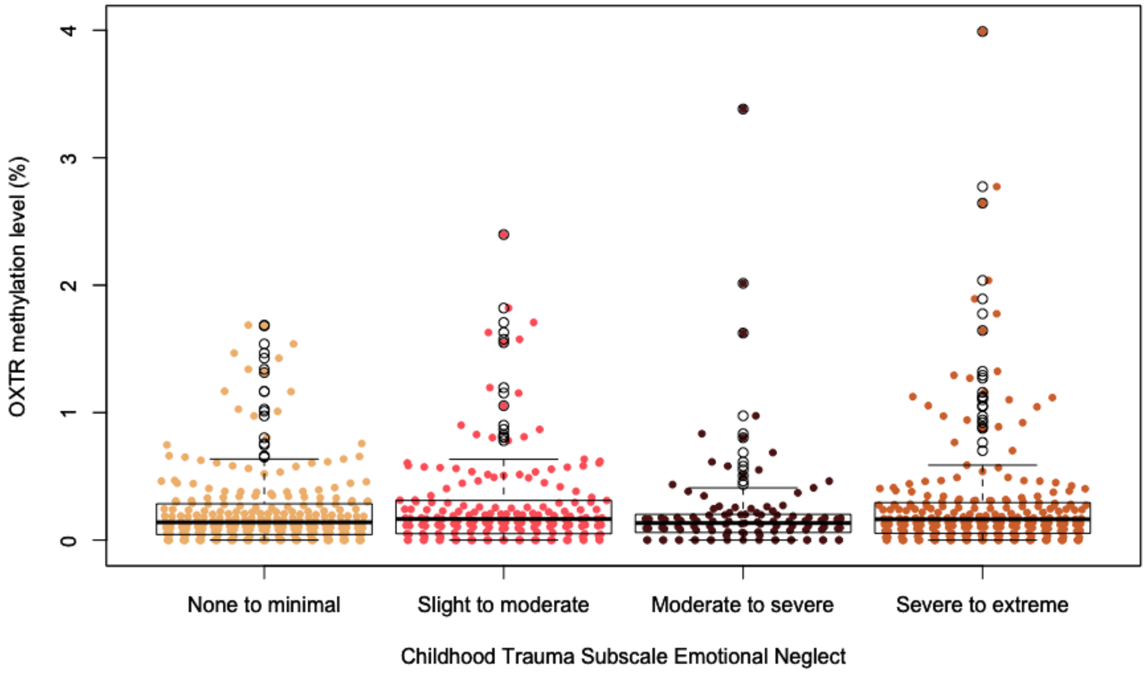

Supplementary Figure 6: Box plots showing oxytocin gene promoter methylation (%) in none to low trauma exposure, low to moderate trauma exposure, moderate to severe trauma exposure and severe to extreme trauma exposure within the CTQ subscale Emotional Neglect. A Kruskall-Wallis nonparametric analysis of variance with pair-wise comparison comparing oxytocin gene promoter methylation (%) between none to low trauma exposure, low to moderate trauma exposure, moderate to severe trauma exposure and severe to extreme trauma exposure within the CTQ subscale Emotional Neglect, showed no significant difference ($H=4.507$, $p = 0.21$, $n = 748$) between the categories. Outliers (detected with Z-transformation) were removed from the graph for a better visualization of the results (whereas within the statistical analyses no outliers were removed).
