# Supplementary material for: Oxytocin receptor gene methylation as a molecular marker for severity of depressive symptoms in affective disorder patients
Source: BMC Psychiatry. 2022 Jun 7;22:381. doi: 10.1186/s12888-022-04031-w (PMC9172116; doi:10.1186/s12888-022-04031-w)
Supplement: Supplementary file 3 — Additional file 3. [file 12888_2022_4031_MOESM3_ESM.pdf]

Additional File 3

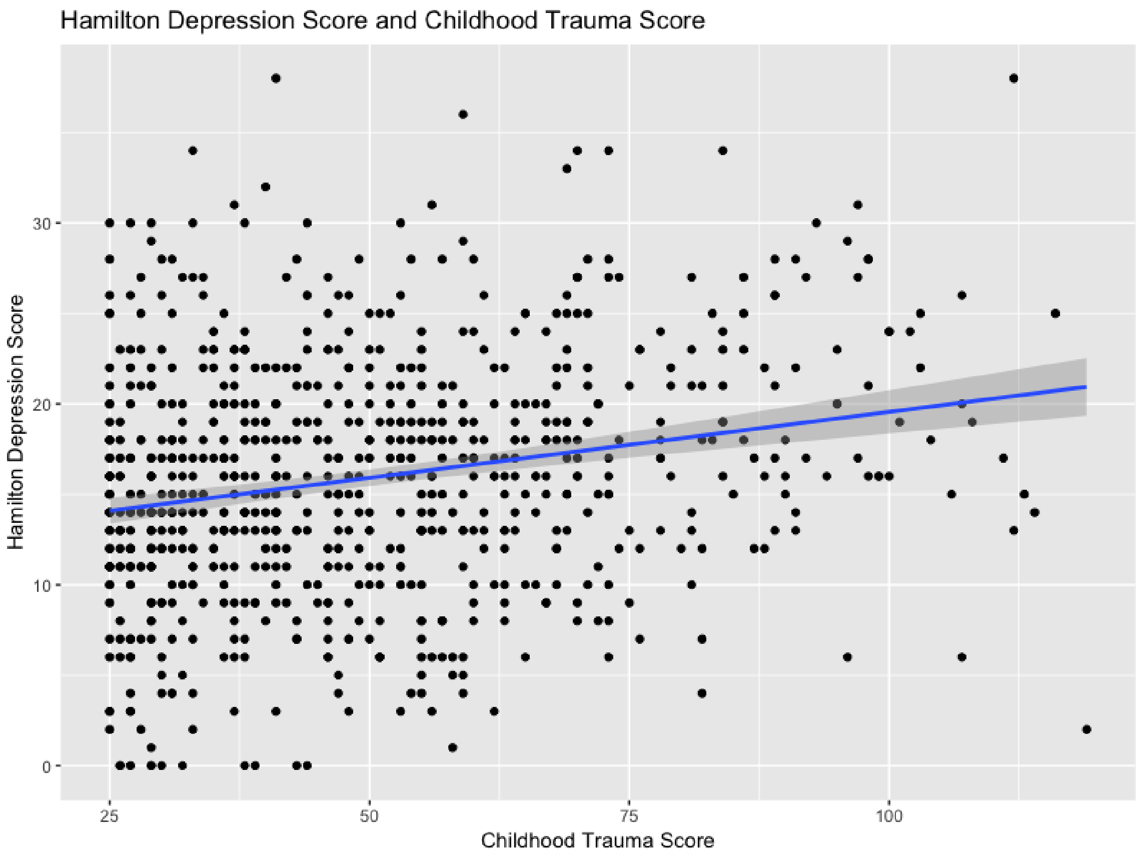

Supplementary Figure 7: Scatterplot of childhood trauma questionnaire (CTQ) with Hamilton Depression Score (HAMD) showing a significant positive correlation (τ =0.15, p<0.0001, n=788).
